# B7-H3 promotes colorectal cancer angiogenesis through activating the NF-κB pathway to induce VEGFA expression

**DOI:** 10.1038/s41419-020-2252-3

**Published:** 2020-01-23

**Authors:** Ruoqin Wang, Yanchao Ma, Shenghua Zhan, Guangbo Zhang, Lei Cao, Xueguang Zhang, Tongguo Shi, Weichang Chen

**Affiliations:** 1 Department of Gastroenterology, The First Affiliated Hospital of Soochow University, 188 Shizi Road, Suzhou, China; 2 Jiangsu Key Laboratory of Clinical Immunology, Soochow University, 708 Renmin Road, Suzhou, China; 3 Jiangsu Institute of Clinical Immunology, The First Affiliated Hospital of Soochow University, 708 Renmin Road, Suzhou, China; 4 Jiangsu Key Laboratory of Gastrointestinal tumor Immunology, The First Affiliated Hospital of Soochow University, 708 Renmin Road, Suzhou, China

**Keywords:** Colorectal cancer, Tumour angiogenesis

## Abstract

Tumor angiogenesis is a hallmark of cancer and is involved in the tumorigenesis of solid tumors. B7-H3, an immune checkpoint molecule, plays critical roles in proliferation, metastasis and tumorigenesis in diverse tumors; however, little is known about the biological functions and molecular mechanism underlying B7-H3 in regulating colorectal cancer (CRC) angiogenesis. In this study, we first demonstrated that the expression of B7-H3 was significantly upregulated and was positively associated with platelet endothelial cell adhesion molecule-1 (CD31) level in tissue samples from patients with CRC. In addition, a series of in vitro and in vivo experiments showed that conditioned medium from B7-H3 knockdown CRC cells significantly inhibited the migration, invasion, and tube formation of human umbilical vein endothelial cells (HUVECs), whereas overexpression of B7-H3 had the opposite effect. Furthermore, B7-H3 promoted tumor angiogenesis by upregulating VEGFA expression. Recombinant VEGFA abolished the inhibitory effects of conditioned medium from shB7-H3 CRC cells on HUVEC angiogenesis, while VEGFA siRNA or a VEGFA-neutralizing antibody reversed the effects of conditioned medium from B7-H3-overexpressing CRC cells on HUVEC angiogenesis. Moreover, we verified that B7-H3 upregulated VEGFA expression and angiogenesis by activating the NF-κB pathway. Collectively, our findings identify the B7-H3/NF-κB/VEGFA axis in promoting CRC angiogenesis, which serves as a promising approach for CRC treatment.

## Facts

- B7-H3 is significantly upregulated and is positively associated with CD31 level in colorectal cancer (CRC) tissue samples.
- B7-H3 modulates tumor angiogenesis by upregulating vascular endothelial growth factor A (VEGFA) expression in CRC cells.
- VEGFA is critical for B7-H3-mediated CRC angiogenesis both in vitro and in vivo.
- B7-H3 promotes VEGFA expression and angiogenesis by activating NF-κB signaling.

## Introduction

Colorectal cancer (CRC) is the third most prevalent cancer worldwide, as well as the third leading cause of cancer-related deaths^1^. With the development of therapeutic methods such as surgical resection, radiotherapy, chemotherapy, immunotherapy, and targeted therapy, the 5-year survival rate of patients with CRC has been significantly improved in recent years^2–4^. However, disease metastasis and relapse are still challenges for CRC clinical therapy^5^. Therefore, it is urgent that we understand the molecular pathogenesis of CRC and identify novel therapeutic targets for CRC treatment.

As a hallmark of cancer, angiogenesis is a critical step in the tumorigenesis of solid cancers^6^. Accumulating evidence has revealed that angiogenesis supplies abundant oxygen and nutrients for tumor cell survival; this process plays a critical role in cancer development, especially in the proliferation and metastasis of CRC cells^7,8^. Anti-angiogenic therapy based on the theory of “starve a tumor to death” has become an attractive strategy against various human malignancies, including CRC^9^. Anti-angiogenic drugs, such as bevacizumab, that target vascular endothelial growth factors (VEGFs) have been approved by the US Food and Drug Administration (FDA) and used in first-line trials with patients with CRC^10^. A growing body of work has indicated that multiple abnormally expressed genes in cancer cells drive vascular growth by attracting and activating endothelial cells. For instance, IL-35 in pancreatic ductal adenocarcinoma cells recruits monocytes via CCL5 and induces macrophages to promote angiogenesis through inducing CXCL1 and CXCL8 expression^11^. The knockdown of SIRT2 significantly decreased angiogenesis by inhibiting the STAT3/VEGFA signaling pathway in CRC cells^12^. NOTCH3 signaling limited tumor angiogenesis independently of the Notch canonical pathway^13^. Nevertheless, the molecular mechanism of the regulation of angiogenesis has not yet been well elucidated.

B7-H3 (also known as CD276), an immune checkpoint molecule found first in 2001, belongs to the B7 superfamily and exerts critical effects on the initiation and termination of immune cell responses by regulating T cell priming, growth, maturation, and tolerance^14^. B7-H3 was found to be overexpressed in a number of solid cancer types, such as CRC, hepatocellular carcinoma, pancreatic cancer, ovarian cancer, and kidney cancer. Apart from its immunologic function, B7-H3 has been reported to be involved in multiple non-immunological functions of tumors, such as proliferation, metastasis, drug resistance, and metabolism^15–17^. A recent study showed that B7-H3 was broadly overexpressed in both cancer cells and tumor vasculature. Ablation of B7-H3-positive tumor cells and tumor vasculature via anti-B7-H3-drug conjugates represents a promising anti-cancer strategy^18^. In pancreatic carcinoma cell lines, soluble B7-H3 strongly promoted invasion and metastasis through activating NF-κB signaling and finally upregulating IL-8 and VEGF expression^19,20^. In contrast, silencing B7-H3 upregulated the mRNA and protein expression of VEGF in the breast cancer cell line MCF7^21^. Based on these findings, B7-H3 plays vital roles in tumor angiogenesis, and its roles in tumor angiogenesis may be dependent on the cellular and tissue contexts. However, the effect of B7-H3 on angiogenesis in CRC is far from completely understood.

In this study, we investigated a positive correlation between B7-H3 levels and microvessel density (MVD) in 125 CRC patients. We further demonstrated that B7-H3 significantly promoted angiogenesis in CRC by using both in vitro and in vivo functional assays. Moreover, we showed that B7-H3 regulated angiogenesis in CRC via activating the NF-κB/VEGFA pathway.

## Materials and methods

### Cell culture

HCT116 and RKO cell lines (ATCC, Manassas, VA, USA) were cultured in DMEM (Biological Industries, BeitHaemek, Israel) supplemented with 10% fetal bovine serum (FBS, Biological Industries) and 1% penicillin-streptomycin (Beyotime, Shanghai, China, #C0222) in a humidified incubator with 5% CO_2_ at 37 °C. The human umbilical vein endothelial cells (HUVECs) were gifted from the Institute for Cardiovascular Science of Soochow University and cultured in EBM-2 medium (Lonza, Walkersville, USA, #CC-3162) supplemented with 10% FBS and 1% penicillin-streptomycin in a humidified incubator with 5% CO_2_ at 37 °C.

### Cell transfection and infection

Three non-overlapping siRNAs for B7-H3 (B7-H3 siRNA-1, B7-H3 siRNA-2, and B7-H3 siRNA-3) and control siRNA were purchased from Shanghai Genechem Co., Ltd. (Shanghai, China). Human VEGFA siRNA and its control siRNA were purchased from Ribobio (Guangzhou, China, #stB0002356C-1–5). Cells were transfected with B7-H3 siRNA, VEGFA siRNA, or control siRNA using Lipofectamine 2000 according to the manufacturer’s protocol. The transfection efficiency was confirmed by the real-time quantitative polymerase chain reaction (RT-qPCR) and western blot.

Lentiviruses carrying the B7-H3 overexpression vector or B7-H3 short hairpin RNA (shRNA) containing the sequence of B7-H3 siRNA-3 were purchased from Shanghai Genechem Co., Ltd. (Shanghai, China). An empty backbone vector was used as a control. Briefly, HCT116 and RKO cells in the exponential growth phase were grown to 30% confluence and infected with lentiviral particles (MOI: 20). After 72 h, the efficiency of overexpression or interference was confirmed by counting GFP-expressing cells under a fluorescence microscope.

### CRC clinical samples and immunohistochemistry (IHC) assay

A total of 125 pairs of CRC samples and adjacent normal tissues were obtained from CRC patients at the First Affiliated Hospital of Soochow University (Suzhou, China). All experiments involving patient specimens were approved by the Institutional Review Board of Soochow University. Informed consent was obtained from each patient. Detailed clinicopathological information is provided in Supplementary Table S1. An IHC assay was conducted as previously described^17^. Briefly, CRC samples or xenograft tumor samples were deparaffinized and rehydrated. After antigen retrieval with 10 mM sodium citrate buffer (pH 6.0), the sections were incubated with goat anti-human 4IgB7-H3 antibody (R&D Systems, MN, USA, #AF1027, 1:100), mouse anti-human CD31 antibody (Abcam, Cambridge, MA, USA, #ab32457, 1:1500), or rabbit anti-human VEGFA antibody (Proteintech, Wuhan, China, #19003–1-AP, 1:500). All sections were then reviewed blindly by two experienced pathologists (Dr. Cao and Dr. Zhan). The scoring criteria for IHC staining were based on the intensity of immunostaining and the percentage of immunoreactive cells as described previously^17^. We calculated the score of the MVD on the basis of previously described criteria^22^.

### Western blot analysis

Cells were harvested and lysed in RIPA buffer (Beyotime, #P0013D) containing protease inhibitors and phosphatase inhibitors (Beyotime, #P1045). Protein concentrations were measured with an Enhanced BCA Protein Assay Kit (Beyotime, #P0010) according to the manufacturer’s instructions. Total protein (30 μg) was separated by 10% SDS-PAGE (Beyotime, #P0012AC) and transferred onto 0.45-µm PVDF membranes (GE Healthcare Life science, Germany). The membranes were blocked with 5% BSA (Fcmacs, Nanjing, China, #FMS-WB021) for 1 h and then incubated with the indicated primary antibodies at 4 °C overnight. The next day, the membranes were then incubated with the corresponding HRP-conjugated secondary antibodies (Beyotime) for 1 h at room temperature. Finally, the membranes were visualized with ECL reagents (NCM Biotech, Suzhou, China, #10100) using a Chemi Doc^TM^ MP Imaging System (Bio-Rad). All antibodies for Western blot analysis are listed in Supplementary Table S2.

### RNA extraction and real-time PCR

Total RNA was extracted from cells using a TransZol Up Plus RNA Kit (Transgen Biotech, Beijing, China, #ER501–01) according to the manufacturer’s instructions. To analyze individual genes, cDNA was synthesized from 1 μg of total RNA using PrimeScript RT Master Mix (Takara, Shiga, Japan, #RR036A) in a volume of 20 μl with the following conditions: 37 °C for 15 min and 85 °C for 5 s. PCR reactions were performed on a CFX96 Touch^TM^ real-time PCR system (Bio-Rad, CA, USA) using SYBR Green Master Mix (Vazyme, Nanjing, China, #Q121–02-AA) according to the manufacturer’s instructions. The cycling conditions were as follows: one cycle at 95 °C for 5 min, 40 cycles of amplification at 95 °C for 10 s, and 60 °C for 30 s. β-actin was used as a constitutive control. All PCRs for each sample were conducted in triplicate. All primers used for RT-qPCR are listed in Supplementary Table S3.

### Enzyme-linked immunosorbent assay (ELISA)

The protein levels of soluble B7-H3 (sB7-H3), VEGFA, fibroblast growth factor 2 (bFGF) and platelet-derived growth factor-BB (PDGF-BB) in cell culture supernatants were measured with ELISA kits (Bright Scistar Biotechnology Co., Ltd., Suzhou, China, #XG-K3004 for soluble B7-H3; NeoBioscience, Shenzhen, China, #EHC108 for VEGFA, #EHC130 for bFGF, and #EHC181 for PDGF-BB) according to the manufacturer’s instructions. To demonstrate the effect of soluble B7-H3 on VEGFA expression, cell culture supernatants of CRC cells treated with or without 50 ng/ml recombinant human B7-H3 (rB7-H3, Sino Biological, Beijing, China, #11188-H02H) for 24 h were collected.

### Conditioned medium

CRC cells (1 × 10^6^) were cultured in 6-well plates overnight, and the culture medium was changed to fresh medium with or without FBS in each well. After 24 h, the conditioned medium was collected and used for ELISA, migration, invasion, and tube formation assays of HUVECs.

### Cell migration and invasion assay

For the migration assay, 500 μl serum-free medium containing 2.5 × 10^4^ HUVECs was added to the 8-μm pore upper chamber in a 24-well plate (BD Biosciences, NJ, USA, #353097), and 750 μl conditioned supernatant with or without 10 ng/ml human/primate VEGF antibody (R&D Systems, #MAB293) or recombinant human VEGF_165_ (Peprotech, Suzhou, China, #100–20) was added to the lower chamber. For the invasion assay, the upper chambers were coated with 100 μl diluted Matrigel (200 μg/ml, Corning, Shanghai, China, #356234) for 2 h. After 16 h of incubation for migration assays or 24 h for invasion assays, HUVECs were fixed with 4% paraformaldehyde for 15 min and stained with crystal violet (Beyotime, #C0121) for 15 min. Finally, the HUVECs on the lower side of the chamber membrane were photographed and counted by an inverted microscope.

### Endothelial tube formation assay

HUVECs (3 × 10^4^) were plated in 96-well plates coated with 50 μl Matrigel (Corning) and cultured in conditioned culture medium for 4 h at 37 °C with 5% CO_2_. Tubules were photographed with a microscope and evaluated by Image-Pro Plus software.

### CCK8 assay

Cell Counting Kit-8 (Dojindo, Kumamoto, Japan #CK04) was used to evaluate cell proliferation. HUVECs cultured with different conditioned media were plated in 96-well plates at a concentration of 5000 cells per well. After 24 h, HUVECs were stained with 10 μl sterile CCK8 solution per well for 4 h at 37 °C. The absorbance was measured at 450 nm as the reference.

### Immunofluorescence

HCT116 and RKO cells (1.5 × 10^5^) were seeded onto coverslips in six-well plates. After 24 h, the cells were fixed with 4% paraformaldehyde and then permeabilized with 1% Triton X-100. Then, the cells were blocked with 1% BSA and treated with a VEGFA (1:1000) antibody overnight. This step was followed by TRITC-labeled goat anti-rabbit secondary antibody (KPL, Gaithersburg, MD, USA) staining for 1 h at 37 °C and DAPI for 5 min. Isotype-matched IgG instead of the primary antibody was used as a negative control. The cells were finally visualized under a confocal laser scanning microscope (Olympus FLUOVIEW FV1000).

### Luciferase reporter assay

NF-κB luciferase reporter plasmids were obtained from Genomeditech (Shanghai, China, #GM-021001P100). For luciferase reporter assay, cells were plated at a density of 1 × 10^5^ cells in a 12-well plate overnight and were transfected with 400 ng of NF-κB luciferase reporter plasmids using Lipofectamine 2000. The cells were washed with ice-cold PBS and lysed in Promega Passive Lysis Buffer (Promega, Madison, USA, #E1910) 24 h after transfection. Then, luciferase activity was examined using a Dual-Luciferase Reporter Assay System (Promega, Madison, USA, #E1910) following the manufacturer’ s instructions.

### Matrigel plug assay in vivo

Six-week-old female BALB/c nude mice were purchased from the Shanghai Laboratory Animal Center. All animal experiments were approved by the Institutional Animal Care and Use Committee of Soochow University (Suzhou, China). All mice were randomly divided into different groups. B7-H3 knockdown or overexpressing HCT116 cells (5 × 10^6^) were suspended at a 1:1 ratio in 200 μL PBS and Matrigel (Corning) and injected into the subcutaneous layer of the right flank of the mice. The tumor volumes were examined every 2 days. To investigate whether the pro-angiogenic effect of B7-H3 was based on NF-κB pathway and VEGFA, mice injected with B7-H3-overexpressing HCT116 cells were treated with BAY11–7082 (MedChemExpress, NJ, USA, #HY-13453) at a dose of 6 mg/kg every other day or bevacizumab (MedChemExpress, NJ, USA, #HY-P9906) at a dose of 1 mg/kg twice per week. To investigate whether B7-H3 blockade and BAY11–7082 or bevacizumab treatment exerts synergetic effects on the inhibition of angiogenesis in vivo, mice injected with HCT116 cells were co-treated with a B7-H3 blocking antibody, 3E8 (Bright Scistar Biotechnology Co., Ltd.) at a dose of 5 mg/kg every other day and BAY11–7082 at a dose of 6 mg/kg every other day or bevacizumab at a dose of 1 mg/kg twice per week. After 2 weeks, the xenografts were harvested. Tumor tissues were embedded in paraffin for IHC. All results of animal experiments were obtained blindly.

### Statistical analysis

All statistical data were analyzed with GraphPad Prism 5.0 (La Jolla, CA, USA). Student’s *t*-test was used to find the differences between two groups. All experiments were performed in triplicate, and *P* < 0.05 was considered statistically significant.

## Results

### Increased B7-H3 expression in tissue samples from CRC patients is positively associated with the microvessel density

**Fig. 1:**
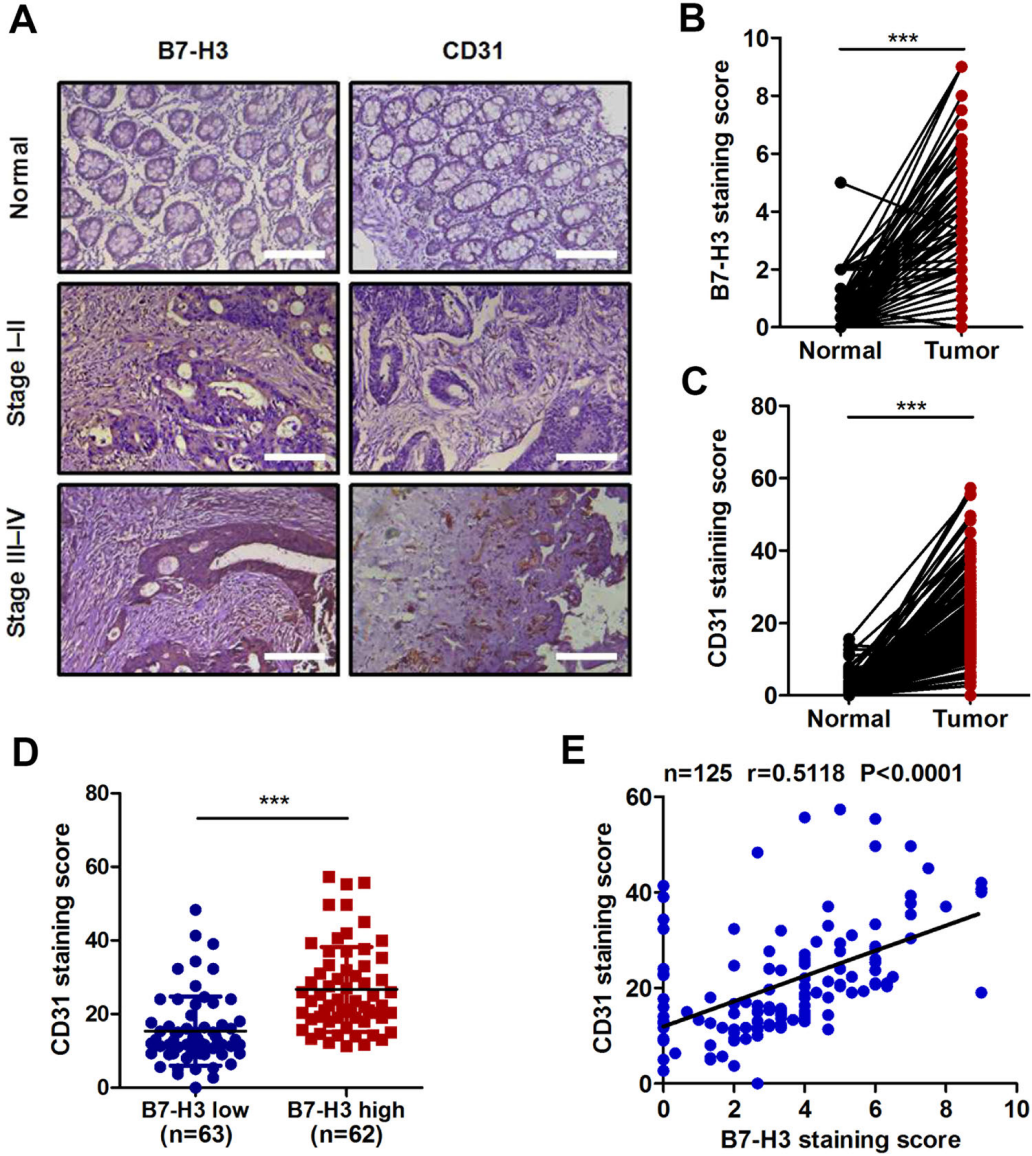
Correlation analysis between B7-H3 and CD31 in tissue samples from CRC patients. **a** Representative images of immunohistochemistry (IHC) for B7-H3 and CD31 in CRC tissues and matched normal tissues from the 125 clinical CRC patients. Scale bar, 100 μm. **b**, **c** B7-H3 (**b**) and CD31 (**c**) protein expression based on their staining index in CRC specimens and matched normal tissues. **d** CD31 protein expression is shown in patients stratified into B7-H3 low (<median value) and B7-H3 high (>median value) groups. **e** Correlation analysis of the staining index of the protein expression levels of B7-H3 and CD31 in human CRC specimens (*n* = 125). The correlation coefficient (r) is shown. The data represent the means ± SEM. ***P < 0.001.

As shown in Fig. 1a, b, the B7-H3 expression level was obviously increased in CRC tissues. As a transmembrane glycoprotein, CD31 is expressed mainly by endothelial cells and various hematopoietic cells and is usually used as a sensitive and specific endothelial marker for MVD^11^. The IHC results showed that the expression of CD31 was obviously higher in CRC tissues than in normal tissues (Fig. 1a, c). In addition, we selected 63 cases (50.4%) with low expression of B7-H3 (<median value) and 62 cases (49.6%) with high expression of B7-H3 (>median value). Patients with high B7-H3 expression in their tumor tissues had significantly higher CD31 expression than patients with low B7-H3 expression (Fig. 1d). More importantly, the expression level of B7-H3 was found to be positively associated with CD31 levels in tissue samples from patients with CRC (Fig. 1e). In addition, the expression of both B7-H3 and CD31 was further increased in advanced (III and IV stages) CRC patients as compared with early stages (I and II) patients (Supplementary Fig. S1a, b). What’ s more, CRC tissue samples with lymph node metastasis showed higher B7-H3 and CD31 expression than those without lymph node metastasis (Supplementary Fig. S1c, d). Together, these clinical data indicate that the upregulation of B7-H3 is closely related to tumor angiogenesis in patients with CRC.

### B7-H3 promotes angiogenesis in vitro

**Fig. 2:**
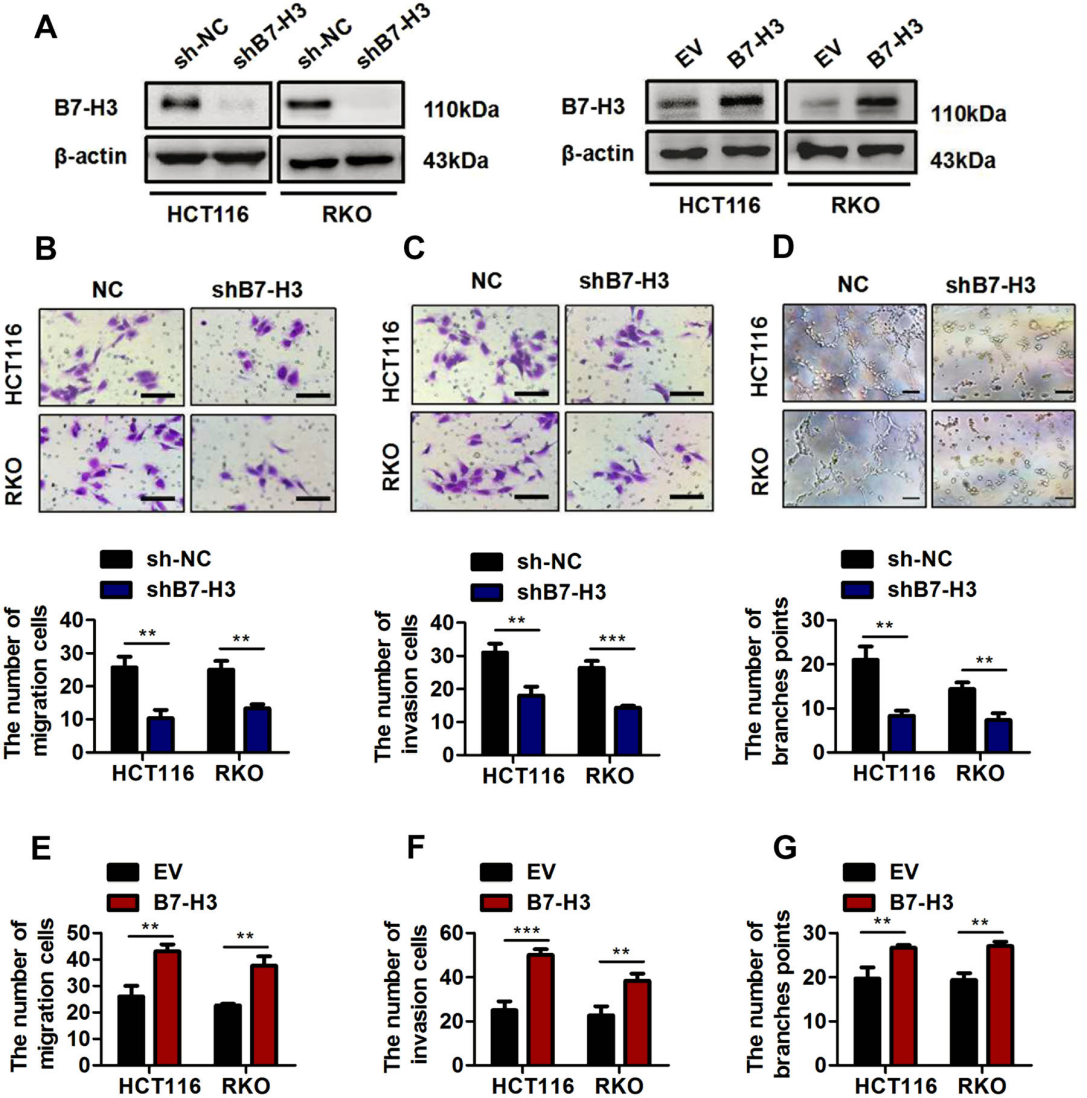
B7-H3 promoted HUVEC migration, invasion and tube formation in vitro. **a** Western blot analysis of B7-H3 in CRC stable cell lines with B7-H3 inhibition (shB7-H3) or their control cell lines (sh-NC) (left). Western blot analysis of B7-H3 in CRC stable cell lines overexpressing B7-H3 (B7-H3) or their control cell lines (EV) (right). β-actin served as a loading control. **b**, **c** Cell migration (**b**) and invasion (**c**) in HUVECs were examined by transwell assays after HUVECs were plated and treated with conditioned medium (CM) from sh-NC cells or shB7-H3 cells. Scale bar, 100 μm. One representative image from three reproducible experiments is shown. Migrated and invaded HUVEC numbers are shown in the bar graph. **d** Effect of CM from sh-NC cells or shB7-H3 cells on tube formation in HUVECs. Scale bar, 100 μm. The number of tubes counted is shown in the bar graph. One representative result from three reproducible experiments is shown. **e**, **f** Cell migration (**e**) and invasion (**f**) in HUVECs were examined by transwell assays after HUVECs were plated and treated with CM from EV cells or B7-H3 cells. Migrated and invaded HUVEC numbers are shown in the bar graph. **g** Effect of CM from EV cells or B7-H3 cells on tube formation in HUVECs. The number of tubes counted is shown in the bar graph. The data represent the means ± SEM. **P < 0.01, ***P < 0.001.

To investigate the function of B7-H3 in CRC angiogenesis, three non-overlapping siRNAs targeting B7-H3 (B7-H3 siRNA-1, B7-H3 siRNA-2, and B7-H3 siRNA-3) were used to knockdown the expression of B7-H3 in CRC cells (Supplementary Fig. S2a). Among the three siRNAs, B7-H3 siRNA-3 had the highest inhibitory effect on B7-H3 expression in CRC cells (Supplementary Fig. S2a). Hence, the sequences of B7-H3 siRNA-3 were chosen to construct the shRNA lentivirus (shB7-H3), which mediated stably inhibiting B7-H3 expression in HCT116 or RKO cells (Fig. 2a, left and Supplementary Fig. S2b, c). Moreover, the ELISA results showed that shB7-H3 lentivirus also reduced the level of soluble B7-H3 in CRC cells (Supplementary Fig. S2d). Because the proliferation and migration of vascular endothelial cells are key steps in angiogenesis, we explored the effect of B7-H3 on the proliferation and migration of HUVECs^23^. As shown in Supplementary Fig. S2e, conditioned medium from shB7-H3 CRC cells had no effect on the cell activity of HUVECs. In addition, the migration and invasion capacity of HUVECs was significantly inhibited by conditioned medium from shB7-H3 CRC cells (Fig. 2b, c). We further investigated whether B7-H3 could promote HUVEC tube formation ability, which involves all steps of angiogenesis^24^. As shown in Fig. 2d, conditioned medium from shB7-H3 CRC cells obviously reduced the number of branch points. In complementary loss-of-function studies, HCT116 or RKO cells stably overexpressing B7-H3 were used (Fig. 2a, right and Supplementary Fig. S3a, b). Besides, the level of soluble B7-H3 was increased in B7-H3-overexpressing CRC cells (Supplementary Fig. S3c). Conditioned medium from B7-H3-overexpressing CRC cells significantly enhanced the migration and invasion capacity and tube formation ability of HUVECs (Fig. 2e–g and Supplementary Fig. S3d-f) but had no effect on the cell activity of HUVECs (Supplementary Fig. S3g). Furthermore, conditioned medium from CRC cells treated with rB7-H3 significantly enhanced the migration and invasion capacity and tube formation ability of HUVECs (Supplementary Fig. S4a-c). These results verified the important effect of B7-H3 on HUVEC angiogenesis.

### B7-H3 promotes the expression of VEGFA in CRC

**Fig. 3:**
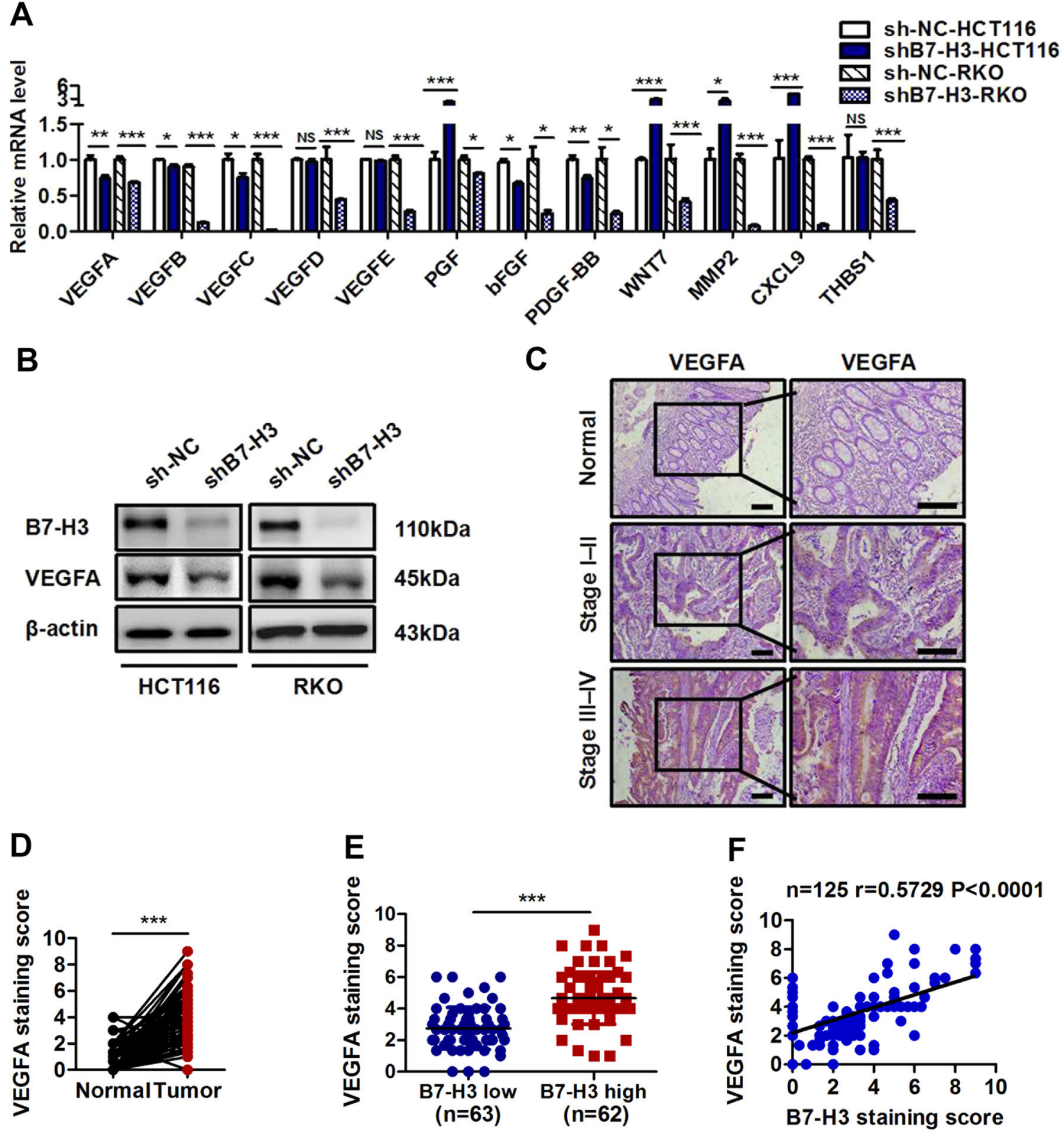
B7-H3 promoted the expression of VEGFA in CRC. **a** The expression of angiogenesis-related genes was detected by RT-qPCR in shB7-H3 HCT116 and RKO cells. **b** Western blot analysis of B7-H3 and VEGFA in the sh-NC and shB7-H3 CRC cell lines. β-actin served as a loading control. **c** Representative images of IHC for VEGFA in CRC tissues and matched normal tissues from the 125 clinical CRC patients. Scale bar, 100 μm. **d** VEGFA protein expression based on the staining index of CRC specimens and matched normal tissues. **e** VEGFA protein expression is shown for patients stratified into B7-H3 low (<median value) and B7-H3 high (>median value) groups. **f** Correlation analysis of the staining index of the protein expression levels of B7-H3 and CD31 in human CRC specimens (*n* = 125). The correlation coefficient (r) is shown. The data represent the means ± SEM. NS no significant difference; *P < 0.05; **P < 0.01; ***P < 0.001.

Given that multiple cytokines and growth factors are involved in tumor angiogenesis^25^, we asked whether B7-H3 promotes angiogenesis by regulating the expression of key angiogenesis-related cytokines. To verify this hypothesis, we first examined the mRNA expression of multiple angiogenesis-related cytokines in CRC cells expressing shB7-H3 or B7-H3 (Fig. 3a and Supplementary Fig. S5a). The mRNA expression of VEGFA, VEGFC, bFGF, and PDGF-BB was significantly downregulated in CRC cells expressing shB7-H3 (Fig. 3a), while it was obviously upregulated in CRC cells overexpressing B7-H3 (Supplementary Fig. S5a). Given that VEGFC is thought to play a primary role in lymphangiogenesis, we analyzed the protein expression of VEGFA, bFGF, and PDGF-BB in CRC cells expressing shB7-H3 or B7-H3. An ELISA assay demonstrated that shB7-H3 significantly reduced the protein concentration of VEGFA, whereas B7-H3 overexpression significantly increased the level of VEGFA in CRC cells (Supplementary Fig. S5b, c). Altered expression of B7-H3 did not affect the protein concentrations of bFGF and PDGF-BB in CRC cells (Supplementary Fig. S5b, c). Furthermore, Western blot results showed that a positive correlation between the level of B7-H3 and VEGFA existed in CRC cells (Fig. 3b and Supplementary Fig. S5d). Furthermore, immunofluorescence (IF) analysis showed consistent results (Supplementary Fig. S5e, f). In addition, we also examined the effect of soluble B7-H3 on VEGFA expression in CRC cells. The ELISA and western blot results revealed that rB7-H3 treatment obviously increased the protein level of VEGFA in CRC cells (Supplementary Fig. S5g, h).

We next investigated the correlation between the level of B7-H3 and VEGFA in CRC clinical samples by IHC staining. We found that the IHC staining score for VEGFA in CRC tissue samples obviously increased compared with that in normal tissues (Fig. 3c, d). In addition, patients with high B7-H3 expression in tumor tissues had significantly higher VEGFA expression than patients with low B7-H3 expression (Fig. 3c, e). Moreover, a positive correlation between VEGFA and B7-H3 expression was found in CRC clinical tissue samples (Fig. 3f). Collectively, these findings suggested that B7-H3 positively regulated VEGFA expression in CRC.

### VEGFA is involved in B7-H3-induced angiogenesis

**Fig. 4:**
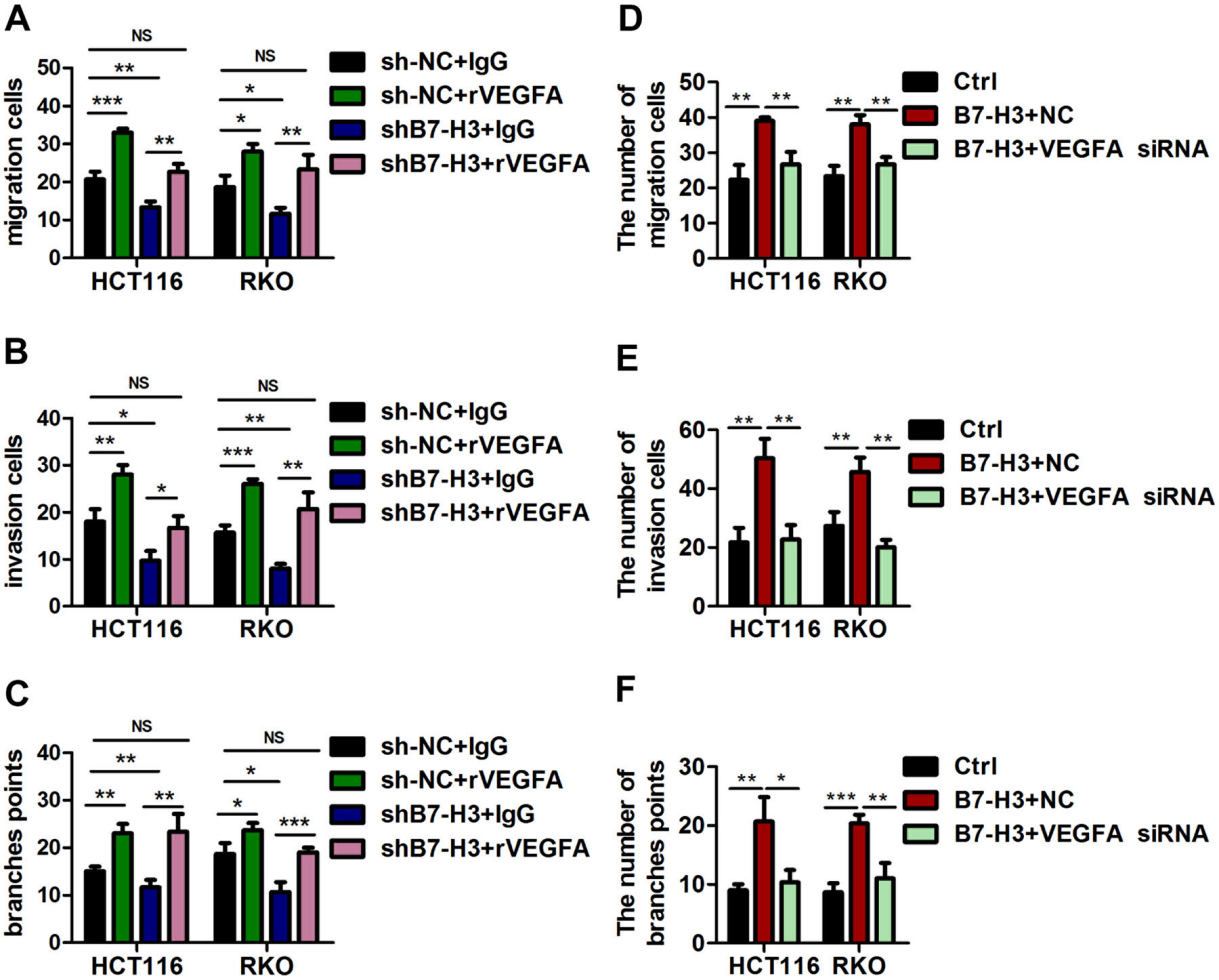
VEGFA was involved in B7-H3-induced angiogenesis. **a**, **b** Cell migration (**a**) and invasion (**b**) in HUVECs were examined by transwell assays after HUVECs were co-treated with CM from sh-NC cells or shB7-H3 cells and IgG or recombinant VEGFA (rVEGFA). Migrated and invaded HUVEC numbers are shown in the bar graph. **c** The tube formation of HUVECs co-treated with CM from sh-NC cells or shB7-H3 cells and IgG or rVEGFA was examined. The number of tubes counted is shown in the bar graph. **d**, **e** Cell migration (**d**) and invasion (**e**) in HUVECs were examined by transwell assays after HUVECs were co-treated with CM from EV cells or B7-H3 cells and siRNA negative control (NC) or VEGFA siRNA. Migrated and invaded HUVEC numbers are shown in the bar graph. **f** The tube formation of HUVECs co-treated with CM from EV cells or B7-H3 cells and NC or VEGFA siRNA was examined. The number of tubes counted is shown in the bar graph. The data represent the means ± SEM. NS no significant difference; *P < 0.05; **P < 0.01; ***P < 0.001.

As B7-H3 positively regulates VEGFA expression in CRC, we wondered whether this important effect of B7-H3 on HUVEC angiogenesis was VEGFA-dependent. We found that treatment with recombinant VEGFA (rVEGFA) abolished the inhibitory effects of conditioned medium from shB7-H3 CRC cells on HUVEC angiogenesis when compared with control IgG treatment or no treatment (Fig. 4a–c and Supplementary Fig. S6a-c). Additionally, we used a commercial siRNA against VEGFA (VEGFA siRNA), which was specific for VEGFA and significantly reduced the mRNA and protein expression levels of VEGFA in CRC cells (Supplementary Fig. S7a-c). Conditioned medium from B7-H3-overexpressing CRC cells treated with VEGFA siRNA obviously reduced HUVEC migration and invasion capacity and tube formation ability, which were enhanced by conditioned medium from B7-H3-overexpressing CRC cells (Fig. 4d–f and Supplementary Fig. S7d-f). Furthermore, the pro-angiogenic effect of conditioned medium from B7-H3-overexpressing CRC cells was reversed by a VEGFA-neutralizing antibody (antiVEGFA) (Supplementary Fig. S7g-i). These results strongly indicate that the promoting effects of B7-H3 on HUVEC angiogenesis were VEGFA-dependent.

### B7-H3 promotes VEGFA expression and angiogenesis dependent on the NF-κB pathway in CRC

**Fig. 5:**
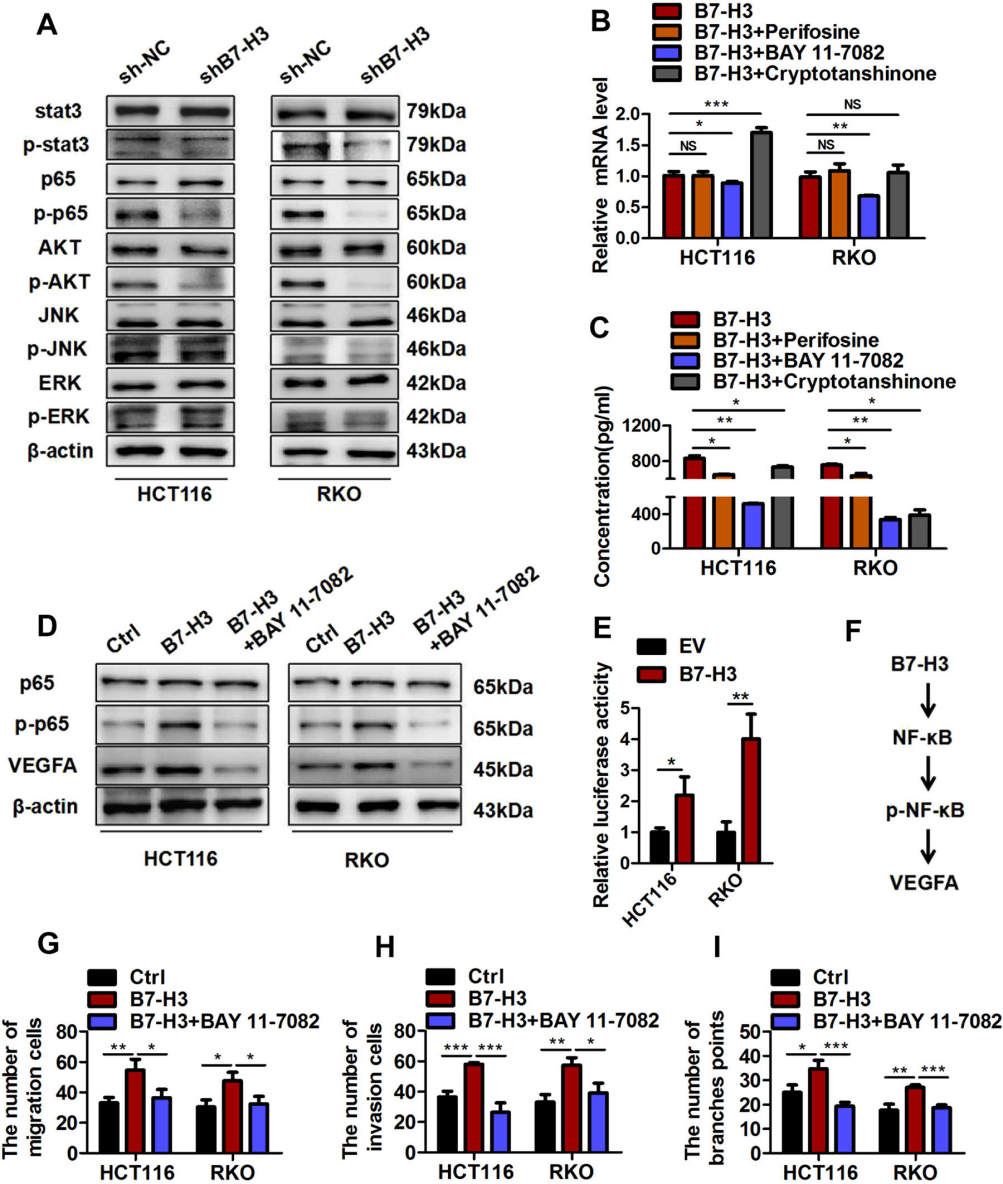
B7-H3 promoted VEGFA expression and angiogenesis through the NF-κB pathway. **a** The expression of p-STAT3, p-p65, p-AKT, p-JNK, and p-ERK1/2 in HCT116 (left) and RKO (right) stable cell lines with B7-H3 inhibition (shB7-H3) or their control cell lines (sh-NC) was analyzed by Western blot. β-actin was used as an internal control. **b** Relative mRNA level of VEGFA in B7-H3 CRC cells after treatment with perifosine, BAY11–7082 or cryptotanshinone were analyzed by RT-qPCR. **c** The protein expression of VEGFA in B7-H3 CRC cells after treatment with perifosine, BAY11–7082 or cryptotanshinone was analyzed by ELISA. **d** The protein expression of VEGFA in B7-H3 CRC cells after treatment with BAY11–7082 was detected by Western blot. **e** NF-κB activity in EV cells or B7-H3 cells was examined by luciferase reporter assay. **f** Schematic representation of the proposed B7-H3/NF-κB/VEGFA axis. **g**, **h** Cell migration (**g**) and invasion (**h**) in HUVECs were examined by transwell assays after HUVECs were co-treated with CM from EV cells or B7-H3 cells and BAY11–7082. Migrated and invaded HUVEC numbers are shown in the bar graph. **i** The tube formation of HUVECs co-treated with CM from EV cells or B7-H3 cells and BAY11–7082 was examined. The number of tubes counted is shown in the bar graph. The data represent the means ± SEM. NS no significant difference; *P < 0.05; **P < 0.01; ***P < 0.001.

Multiple signaling pathways, such as ERK, JNK, AKT, NF-κB, and STAT3, have been reported to be involved in regulating the expression of VEGFA^26–29^. In addition, previous studies indicated that ERK, JNK, AKT, NF-κB, and STAT3 signaling are the downstream targets of B7-H3 in tumors^19,30,31^. Hence, we hypothesized that B7-H3 could regulate the expression of VEGFA in CRC via one or more of these signaling pathways. To test our hypothesis, we examined the activity of the abovementioned signaling pathways (determined by the levels of p-ERK, p-JNK, p-AKT, p-p65, and p-STAT3) in HCT116 and RKO cells treated with shRNA-B7-H3. As shown in Fig. 5a, western blot results revealed that B7-H3 knockdown obviously reduced the phosphorylation levels of AKT, NF-κB, and STAT3 in both HCT116 and RKO cells. Furthermore, an AKT inhibitor (perifosine), NF-κB inhibitor (BAY11–7082), and STAT3 inhibitor (cryptotanshinone) were used to investigate whether activated AKT, NF-κB, or STAT3 upregulated VEGFA expression. RT-qPCR and ELISA results demonstrated that BAY11–7082 treatment reduced both the mRNA and protein levels of VEGFA in B7-H3-overexpressing CRC cells compared with the other two inhibitors (Fig. 5b, c). Moreover, western blot results showed that increased levels of p-p65 and VEGFA were found in B7-H3-overexpressing CRC cells, whereas BAY11–7082 treatment significantly decreased the expression of p-p65 and VEGFA in both HCT116 and RKO cells overexpressing B7-H3 (Fig. 5d). A luciferase reporter assay showed that B7-H3 overexpression increased the NF-κB activity (Fig. 5e). Furthermore, B7-H3 knockdown inhibited, B7-H3 overexpression promoted, the expression of NF-κB target genes, such as IL-8, Bcl2, cyclin D1, and COX-2 in both HCT116 and RKO cells (Supplementary Fig. S8a, b). Besides, rB7-H3 treatment significantly increased the levels of p-p65 and VEGFA in CRC cells (Supplementary Fig. S8c and Supplementary Fig. S5h). Our results suggest that B7-H3 positively promotes VEGFA expression through the NF-κB pathway (Fig. 5f).

To further examine the role of the NF-κB signaling pathway in B7-H3-mediated tumor angiogenesis, we examined the migration and invasion capacity and tube formation ability of HUVECs treated with conditioned medium from B7-H3-overexpressing CRC cells treated with or without BAY11–7082. As expected, the enhanced pro-angiogenic ability of HUVECs incubated with conditioned medium from B7-H3-overexpressing CRC cells was reversed by the addition of BAY11–7082 (Fig. 5g–i and Supplementary Fig. S8d-f). Taken together, above results strongly indicated that B7-H3 promoted tumor angiogenesis in a manner mainly dependent on the NF-κB/VEGFA signaling pathway in CRC.

### B7-H3 promotes angiogenesis in Matrigel plugs in vivo

**Fig. 6:**
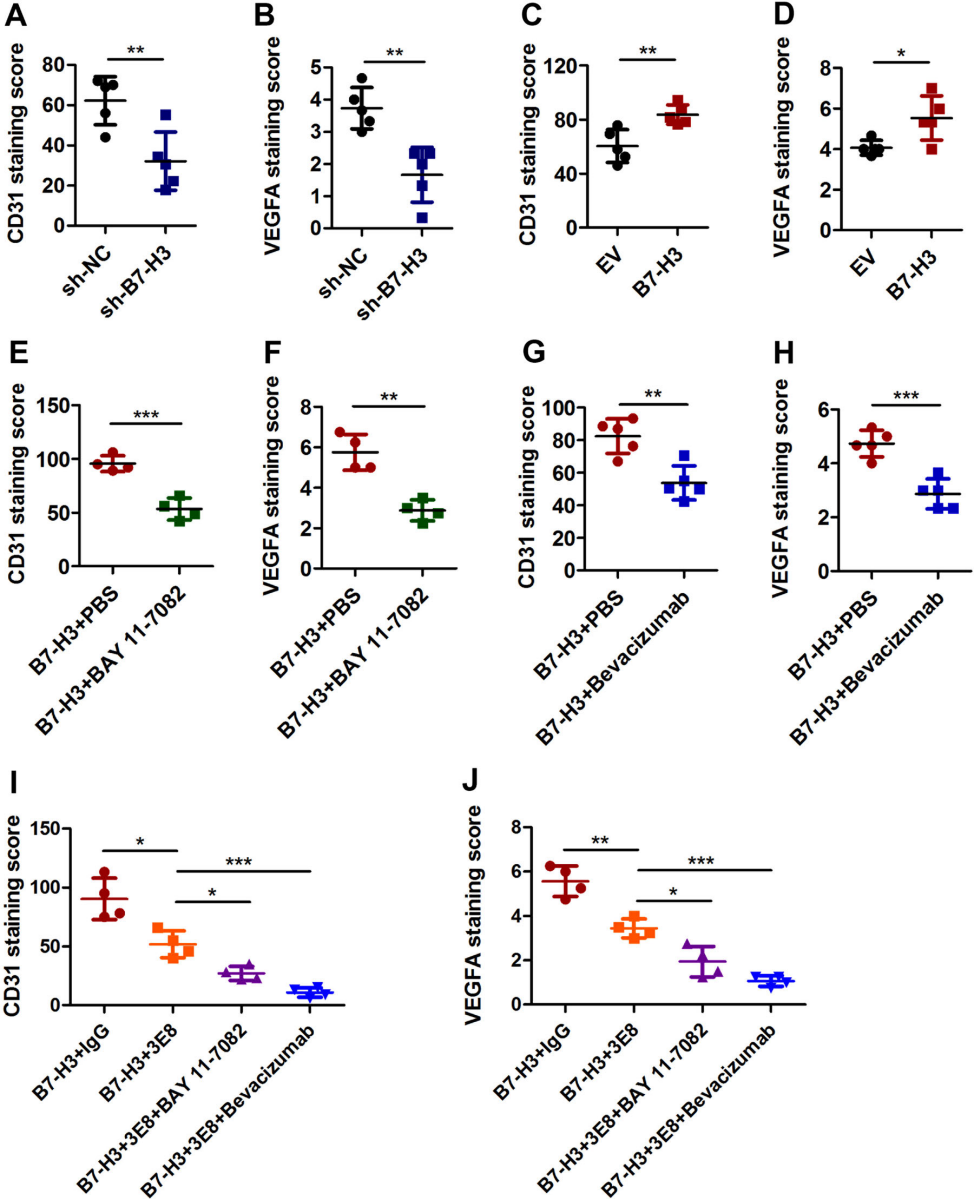
B7-H3 promoted angiogenesis via NF-κB/VEGFA pathway in Matrigel plugs in vivo. **a**, **b** CD31 (**a**) and VEGFA (**b**) protein expression based on their IHC staining index results in subcutaneous tumors formed by sh-NC-HCT116 and shB7-H3-HCT116 cells. *N* = 5. **c**, **d** CD31 (**c**) and VEGFA (**d**) protein expression based on their IHC staining index results in subcutaneous tumors formed by EV-HCT116 and B7-H3-HCT116 cells. *N* = 5. **e**, **f** CD31 (**e**) and VEGFA (**f**) protein expression based on their IHC staining index results in subcutaneous B7-H3-HCT116 tumors and B7-H3-HCT116 tumors treated with BAY11–7082 (6 mg/kg). **g**, **h** CD31 (**g**) and VEGFA (**h**) protein expression based on their IHC staining index results in subcutaneous B7-H3-HCT116 tumors and B7-H3-HCT116 tumors treated with bevacizumab (1 mg/kg). **i**, **j** CD31 (**i**) and VEGFA (**j**) protein expression based on their IHC staining index results in subcutaneous B7-H3-HCT116 tumors and B7-H3-HCT116 tumors co-treated with 3E8 (5 mg/kg) and BAY11–7082 (6 mg/kg) or bevacizumab (1 mg/kg). *N* = 5. The data represent the means ± SEM. *P < 0.05; **P < 0.01; ***P < 0.001.

The above results indicated that B7-H3 positively affects CRC angiogenesis via NF-κB/VEGFA signaling pathway. To verify the mechanism in vivo, Matrigel plug xenograft models of B7-H3 shRNA HCT116 tumors and B7-H3-overexpressing HCT116 tumors were established in nude mice. Although B7-H3 knockdown or overexpression had no significant effect on tumor size or tumor weight (Supplementary Fig. S9a-f), knockdown of B7-H3 in HCT116 cells significantly decreased the MVD and inhibited VEGFA expression in Matrigel plug tumors compared with the control conditions, while overexpression of B7-H3 showed the opposite results (Fig. 6a–d and Supplementary Fig. S9g, h). To determine whether B7-H3-mediated pro-angiogenesis was NF-κB/VEGFA pathway-dependent in vivo, BAY11–7082 or bevacizumab were administered to the mice injected with B7-H3-overexpressing HCT116 cells. Both BAY11–7082 and bevacizumab treatment significantly reduced the sizes and weight of tumors derived from B7-H3-overexpressing HCT116 cells (Supplementary Fig. S10a-d). More importantly, BAY11–7082 or bevacizumab treatment reversed the promotion of B7-H3 overexpression on the MVD and VEGFA expression in vivo (Fig. 6e–h, Supplementary Fig. S10e, f). Taken together, B7-H3 promotes angiogenesis by regulating NF-κB/VEGFA signaling pathway in vivo.

As B7-H3 promoted tumor angiogenesis in CRC via the NF-κB/VEGFA signaling pathway, we wondered whether B7-H3 blockade and BAY11–7082 or bevacizumab co-treatment exerts synergetic effects on the inhibition of angiogenesis in vivo. Hence, we co-treated the mice injected with B7-H3-overexpressing HCT116 cells with 3E8, a specific blocking antibody for B7-H3, and BAY11–7082 or bevacizumab. The results of tumor size and weight showed that co-treatment with 3E8 and BAY11–7082 or bevacizumab obviously inhibited the tumor growth as compared with IgG or 3E8 treatment (Supplementary Fig. S10g, h). More importantly, co-treatment with 3E8 and BAY11–7082 or bevacizumab had a more inhibitory effect on the MVD and VEGFA expression than IgG or 3E8 treatment (Fig. 6i, j, Supplementary Fig. S10i). These results demonstrated that co-treatment with 3E8 and BAY11–7082 or bevacizumab exerted a synergetic effect in the treatment of CRC.

## Discussion

B7-H3 is overexpressed in multiple malignant tumors, including CRC, and is positively associated with advanced tumor-node-metastasis (TNM) stage and poor prognosis^32–35^. Our results also showed that the level of B7-H3 was much higher in CRC tissue samples as compared with normal tissues. The expression of B7-H3 could be regulated at the transcriptional and post-transcriptional levels in cancer cells. It has been reported that B7-H3 was directly targeted by miR-506 in mantle cell lymphoma^36^; by miR-29 in medulloblastoma^37^; and by miR-143 in CRC^38^. Zhang et al. found that immunoglobulin-like transcript 4 (ILT4) drove B7-H3 expression via PI3K/AKT/mTOR signaling in non-small cell lung cancer^39^. In addition, B7-H3 was transcriptionally regulated by BRD4 in pancreatic cancer cell^40^. Therefore, the regulatory mechanism of B7-H3 expression in CRC might be complicated and should trigger further investigation. Mounting evidence has revealed that B7-H3 is involved in the progression and metastasis of CRC, suggesting that B7-H3 has become a new potential prognostic marker and therapeutic target for CRC^32^. Nowadays, it is believed that an immune checkpoint inhibitor (ICI) plus anti-angiogenesis would become a promising strategy, overcoming treatment resistance and improving patients’ prognosis^41^. However, the role and molecular mechanism of B7-H3 in regulating CRC angiogenesis remain unclear. A prior study has shown that the expression of B7-H3 is strikingly different between physiological and pathological angiogenic vessels and is significantly upregulated in the blood vessels of various human cancers^42^. A pyrrolobenzodiazepine-conjugated anti-B7-H3-drug could destroy both cancer cells and tumor vasculature and dramatically improved survival in mouse models with B7-H3-expressing tumors^18^. In addition, soluble B7-H3 strongly upregulated IL-8 and VEGF expression in pancreatic carcinoma cells, while silencing B7-H3 upregulated the mRNA and protein expression of VEGF in the breast cancer cell line MCF7^21^. Herein, our study demonstrated that the expression level of B7-H3 was increased and positively associated with CD31 level in tissue samples from patients with CRC. The most striking finding of our research was that conditioned medium from shB7-H3 CRC cells significantly inhibited the migration, invasion and tube formation of HUVECs in vitro. Moreover, the MVD obviously decreased in shB7-H3 HCT116 Matrigel plug tumors in nude mice. In contrast, B7-H3 overexpression in CRC cells promoted angiogenesis in vitro and in vivo. Additionally, conditioned medium from CRC cells treated with rB7-H3 also promoted the migration, invasion and tube formation of HUVECs in vitro. Taken together, these results indicate that B7-H3 may play a critical role in the regulation of angiogenesis in CRC.

Angiogenesis is an essential component of the tumor metastatic pathway^43,44^. Zhu et al. noted that the MVD was positively associated with invasion of depth, lymph node metastasis, distant metastasis and TNM stages in CRC patients, suggesting that MVD may represent a promising metastatic and prognostic biomarker for CRC^45^. In invasive ductal carcinomas of the breast, the MVD was also associated with tumor size and lymph node metastasis^46^. Moreover, the MVD was significantly higher in laryngeal carcinoma patients with metastasis than in laryngeal carcinoma patients without metastasis^47^. In this study, our results showed that the expression of CD31, a sensitive and specific endothelial marker for MVD, was positively associated with TNM stages and lymph node metastasis. These results indicated that angiogenesis exerts important effects on metastasis in multiple tumors, including CRC. An increasing number of studies noted that multiple genes were involved in the angiogenesis-mediated tumor metastasis. For example, Tie1 deletion prevented extravasation of tumor cells into the lungs and reduced metastatic foci by inhibiting angiogenesis and vascular abnormalization in experimental murine metastasis models^48^. Exosomal miR-25–3p, derived from CRC cells, promoted pre-metastatic niche formation by inducing vascular permeability and angiogenesis^49^. In the current study, we demonstrated that B7-H3 was an important oncogene involved in CRC angiogenesis. More importantly, a growing body of evidence showed that B7-H3 could affect the adhesion, migration, invasion, and metastasis of cancer cells^50^. A previous study showed that B7-H3 overexpression promoted cell migration and invasion through the regulation of PI3K/Akt/STAT3 signaling pathway in human bladder cancer^51^. Additionally, B7-H3 was reported to induce the epithelial-mesenchymal transition, which promoted CRC cell invasion and metastasis^52^. Herein, we found that the expression of B7-H3 was positively associated with TNM stage and lymph node metastasis in the tissue samples of patients with CRC. Given that B7-H3 exerted important roles in angiogenesis and metastasis, we speculated that B7-H3 probably took part in the metastasis through promoting angiogenesis in CRC. Therefore, it may be valuable to explore the effect of B7-H3-mediated angiogenesis on metastasis of CRC in our future study.

Tumor angiogenesis is regulated mainly by a series of cytokines and growth factors produced by various types of cells, including cancer cells in the tumor microenvironment^25^. Herein, we analyzed the mRNA expression level of a series of key angiogenesis-related cytokines including VEGFA, VEGFB, VEGFC, VEGFD, VEGFE, PGF, bFGF, PDGF-BB, WNT7, MMP-2, CXCL9, and THBS1^25^. Among these angiogenesis-related cytokines, the mRNA expression of VEGFA, VEGFC, bFGF, and PDGF-BB was positively associated with B7-H3 level in both HCT116 and RKO cells. Given that VEGFC is thought to play a primary role in lymphangiogenesis, the protein expression of VEGFA, bFGF, and PDGF-BB in CRC cells expressing shB7-H3 or B7-H3 was analyzed by ELISA. The results showed that altered expression of B7-H3 could only affect the protein concentrations of VEGFA in CRC cells. Therefore, we concluded that B7-H3 positively regulated VEGFA expression in CRC. The results of western blot and IF indicated that the protein expression levels of VEGFA were significantly decreased in B7-H3 knockdown CRC cells. Moreover, the level of VEGFA was obviously higher in CRC tissue samples than in normal tissues. There was a positive correlation between VEGFA and B7-H3 expression in CRC clinical tissue samples. In addition, we also revealed that rB7-H3 treatment obviously increased the VEGFA protein level in CRC cells. Our data imply that B7-H3 may promote angiogenesis by upregulating the expression of VEGFA in CRC.

As a type of endothelial cell-specific mitogen, VEGFA has been reported to induce physiological and pathological angiogenesis by activating various signaling pathways that promote endothelial cell growth, migration, differentiation, and vascular permeability^53,54^. Previous studies indicated that the expression of VEGF was increased in CRC clinical tissue samples and correlated with a poor clinical outcome^55,56^. Lin et al. noted that DHX32, a novel member of the DEAH family, enhanced the angiogenesis and growth of CRC by inducing VEGFA expression^57^. Silencing SIRT2 significantly suppressed tumor angiogenesis by inhibiting the STAT3/VEGFA signaling pathway in CRC cells^12^. Additionally, Grb2-associated binder 2 (Gab2) promoted intestinal tumor growth and angiogenesis by upregulating VEGF expression in a MEK/ERK/c-Myc pathway-dependent manner^58^. Furthermore, bevacizumab, a humanized monoclonal antibody against VEGFA, has been approved by the US FDA for use in combination with chemotherapy in patients with advanced CRC^7,59^. Hence, VEGFA exerts a pivotal role in the angiogenesis of CRC and has become a predominant target for anti-angiogenic drugs^60^. In the present study, we found that recombinant VEGFA treatment abolished the inhibitory effects of conditioned medium from shB7-H3 CRC cells on HUVEC angiogenesis, while VEGFA siRNA or a VEGFA-neutralizing antibody reversed the effects of conditioned medium from B7-H3-overexpressing CRC cells on HUVEC angiogenesis. Moreover, bevacizumab treatment obviously reversed the effect of B7-H3 on tumor angiogenesis in vivo. These findings clearly demonstrate that the important effect of B7-H3 on CRC angiogenesis is VEGFA-dependent.

Previous studies indicated that multiple signaling pathways, such as ERK, JNK, AKT, NF-κB, and STAT3, were involved in regulating the expression of VEGFA^26–29^. Thus, we examined the activity of the abovementioned signaling pathways, which have been indicated as the downstream signaling pathways of B7-H3 in tumors^19,30,31^. Our results revealed that B7-H3 knockdown obviously reduced the phosphorylation levels of AKT, NF-κB, and STAT3 in both HCT116 and RKO cells. Next, we used inhibitors against the AKT (perifosine), NF-κB (BAY11–7082), and STAT3 (cryptotanshinone) pathways to determine which pathway is most important in regulating VEGFA expression. The results demonstrated that the NF-κB pathway, rather than the AKT pathway or the STAT3 pathway, had a major effect on B7-H3-induced VEGFA expression in CRC cells. Furthermore, the luciferase reporter assay results showed that B7-H3 overexpression increased the NF-κB activity. Additionally, rB7-H3 treatment significantly increased the levels of p-p65 and VEGFA in CRC cells. These results suggest that B7-H3 promotes VEGFA expression dependent on the NF-κB pathway in CRC. Due to the technical limitation, at the current stage we could not completely clarify the precise way of B7-H3 to activate the NF-κB pathway in CRC. Further investigations are required to answer this question. The NF-κB signaling pathway has been reported to regulate physiological and pathological processes in CRC, such as proliferation, metastasis, and angiogenesis^61,62^. Importantly, the NF-κB pathway regulates the expression of many tumor angiogenesis-associated genes, such as VEGF, PDGF-BB, MMP-2, and MMP-9^63,64^. Herein, we found that BAY11–7082 significantly reversed the enhanced pro-angiogenic ability of HUVECs incubated with conditioned medium from B7-H3-overexpressing CRC cells. Moreover, BAY11–7082 treatment obviously reversed the effect of B7-H3 on tumor angiogenesis in vivo.

As described above, BAY11–7082 or bevacizumab treatment could reverse the effect of B7-H3 on CRC angiogenesis in vitro and in vivo. These results provide a rationale for the combination therapy of B7-H3 inhibitors with BAY11–7082 or bevacizumab in CRC. As expected, co-treatment with 3E8, a specific blocking antibody for B7-H3, and BAY11–7082 or bevacizumab obviously inhibited the tumor growth. More importantly, co-treatment with 3E8 and BAY11–7082 or bevacizumab had a more inhibitory effect on the MVD and VEGFA expression in vivo. Therefore, our results demonstrate that combination therapy of B7-H3 inhibitors with BAY11–7082 or bevacizumab may be an effective therapeutic strategy for CRC.

In summary, we demonstrated the important function of B7-H3 in CRC angiogenesis in vitro and in vivo. Furthermore, our results revealed that B7-H3 promoted tumor angiogenesis by upregulating VEGFA expression in an NF-κB pathway-dependent manner (Supplementary Fig. S11). Therefore, a therapeutic strategy based on the inhibition of B7-H3 to disrupt tumor angiogenesis may be a promising approach for CRC treatment.

## Supplementary information

Supplementary Figure legends

Supplementary Table S1.

Supplementary Table S2.

Supplementary Table S3.

Supplementary Figure S1.

Supplementary Figure S2.

Supplementary Figure S3.

Supplementary Figure S4.

Supplementary Figure S5.

Supplementary Figure S6.

Supplementary Figure S7.

Supplementary Figure S8.

Supplementary Figure S9.

Supplementary Figure S10.

Supplementary Figure S11.

DECLARATION OF CONTRIBUTIONS TO ARTICLE

Reproducibility Checklist

## References

[1] Siegel RL, Miller KD, Jemal A (2019). Cancer statistics, 2019. CA Cancer J. Clin..

[2] Mármol Inés, Quero Javier, Rodríguez-Yoldi María Jesús, Cerrada Elena (2019). Gold as a Possible Alternative to Platinum-Based Chemotherapy for Colon Cancer Treatment. Cancers.

[3] Ganesh K (2019). Immunotherapy in colorectal cancer: rationale, challenges and potential. Nat. Rev. Gastroenterol. Hepatol..

[4] Rotte A (2019). Combination of CTLA-4 and PD-1 blockers for treatment of cancer. J. Exp. Clin. Cancer Res..

[5] Smith JJ, Deane NG, Dhawan P, Beauchamp RD (2008). Regulation of metastasis in colorectal adenocarcinoma: a collision between development and tumor biology. Surgery.

[6] P B (1995). Angiogenesis in colorectal tumors: microvessel quantitation in adenomas and carcinomas with clinicopathological correlations. Cancer Res..

[7] Weis SM, Cheresh DA (2011). Tumor angiogenesis: molecular pathways and therapeutic targets. Nat. Med..

[8] Mihalache A, Rogoveanu I (2014). Angiogenesis factors involved in the pathogenesis of colorectal cancer. Curr. Health Sci. J..

[9] Rivera LB, Gabriele B (2015). CANCER. Tumor angiogenesis, from foe to friend. Science.

[10] LB S (2008). Bevacizumab in combination with oxaliplatin-based chemotherapy as first-line therapy in metastatic colorectal cancer: a randomized phase III study. J. Clin. Oncol. Off. J. Am. Soc. Clin. Oncol..

[11] Huang C (2018). Interleukin 35 expression correlates with microvessel density in pancreatic ductal adenocarcinoma, recruits monocytes, and promotes growth and angiogenesis of xenograft tumors in mice. Gastroenterology.

[12] Hu F (2018). Inhibition of SIRT2 limits tumour angiogenesis via inactivation of the STAT3/VEGFA signalling pathway. Cell Death Dis..

[13] Lin S (2017). Non-canonical NOTCH3 signalling limits tumour angiogenesis. Nat. Commun..

[14] Chapoval AI (2001). B7-H3: A costimulatory molecule for T cell activation and IFN-[gamma] production. Nat. Immunol..

[15] Nygren MK, Tekle C, Ingebrigtsen VA, Fodstad O (2011). B7-H3 and its relevance in cancer; immunological and non-immunological perspectives. Front Biosci..

[16] Kang FB (2015). B7-H3 promotes aggression and invasion of hepatocellular carcinoma by targeting epithelial-to-mesenchymal transition via JAK2/STAT3/Slug signaling pathway. Cancer Cell Int..

[17] Shi T (2019). B7-H3 promotes aerobic glycolysis and chemoresistance in colorectal cancer cells by regulating HK2. Cell Death Dis..

[18] Seaman S (2017). Eradication of tumors through simultaneous ablation of CD276/B7-H3-positive tumor cells and tumor vasculature. Cancer Cell.

[19] Xie C (2016). Soluble B7-H3 promotes the invasion and metastasis of pancreatic carcinoma cells through the TLR4/NF-kappaB pathway. Sci. Rep..

[20] Dong P, Xiong Y, Yue J, Hanley SJB, Watari H (2018). B7H3 as a promoter of metastasis and promising therapeutic target. Front Oncol..

[21] Sun J (2014). B7-H3 expression in breast cancer and upregulation of VEGF through gene silence. Onco Targets Ther..

[22] Weidner N (1995). Current pathologic methods for measuring intratumoral microvessel density within breast carcinoma and other solid tumors. Breast Cancer Res. Treat..

[23] Hoeben A (2004). Vascular endothelial growth factor and angiogenesis. Pharm. Rev..

[24] Ahmed Z, Bicknell R (2009). Angiogenic signalling pathways. Methods Mol. Biol..

[25] De Palma M, Biziato D, Petrova TV (2017). Microenvironmental regulation of tumour angiogenesis. Nat. Rev. Cancer.

[26] Wu G (2006). Involvement of COX-2 in VEGF-induced angiogenesis via P38 and JNK pathways in vascular endothelial cells. Cardiovasc Res..

[27] Lei Z (2018). PARK2 inhibits osteosarcoma cell growth through the JAK2/STAT3/VEGF signaling pathway. Cell Death Dis..

[28] Wang Z, Jin C, Li X, Ding K (2019). Sulfated polysaccharide JCS1S2 inhibits angiogenesis via targeting VEGFR2/VEGF and blocking VEGFR2/Erk/VEGF signaling. Carbohydr. Polym..

[29] Zhu CC (2018). CCR6 promotes tumor angiogenesis via the AKT/NF-kappaB/VEGF pathway in colorectal cancer. Biochim Biophys. Acta Mol. Basis Dis..

[30] Y L (2017). B7-H3 promotes gastric cancer cell migration and invasion. Oncotarget.

[31] Zhang P, Chen Z, Ning K, Jin J, Han X (2017). Inhibition of B7-H3 reverses oxaliplatin resistance in human colorectal cancer cells. Biochem Biophys. Res. Commun..

[32] Ingebrigtsen VA (2012). B7-H3 expression in colorectal cancer: nuclear localization strongly predicts poor outcome in colon cancer. Int. J. Cancer.

[33] Sun J (2010). Clinical significance and regulation of the costimulatory molecule B7-H3 in human colorectal carcinoma. Cancer Immunol. Immunother..

[34] L C (2016). Assessment of combined expression of B7-H3 and B7-H4 as prognostic marker in esophageal cancer patients. Oncotarget.

[35] E P, KC O, X Z (2016). Molecular pathways: targeting B7-H3 (CD276) for human cancer immunotherapy. Clin. Cancer Res. Off. J. Am. Assoc. Cancer Res..

[36] XW Z (2019). MicroRNA-506 inhibits the proliferation and invasion of mantle cell lymphoma cells by targeting B7H3. Biochem. Biophys. Res. Commun..

[37] IJ, P. et al. Role of MYC-miR-29-B7-H3 in medulloblastoma growth and angiogenesis. *J. Clin. Med.* **8**, pii:E1158 (2019). 10.3390/jcm8081158 PMC6723910 31382461

[38] X Z (2016). TGF-β1 promotes colorectal cancer immune escape by elevating B7-H3 and B7-H4 via the miR-155/miR-143 axis. Oncotarget.

[39] P Z (2015). ILT4 drives B7-H3 expression via PI3K/AKT/mTOR signalling and ILT4/B7-H3 co-expression correlates with poor prognosis in non-small cell lung cancer. FEBS Lett..

[40] J Z (2019). B7-H3 is regulated by BRD4 and promotes TLR4 expression in pancreatic ductal adenocarcinoma. Int. J. Biochem. Cell Biol..

[41] M Y (2019). Synergistic effect of immune checkpoint blockade and anti-angiogenesis in cancer treatment. Mol. Cancer.

[42] Seaman S (2007). Genes that distinguish physiological and pathological angiogenesis. Cancer Cell.

[43] BR Z (1998). Angiogenesis and tumor metastasis. Annu. Rev. Med..

[44] DR B, BR Z (2015). The contribution of angiogenesis to the process of metastasis. Cancer J. (Sudbury, Mass).

[45] B Z (2017). Evaluation of the correlation of vasculogenic mimicry, ALDH1, KAI1 and microvessel density in the prediction of metastasis and prognosis in colorectal carcinoma. BMC Surg..

[46] E Ş, S Ş, C G (2016). Comparison of microvessel density with prognostic factors in invasive ductal carcinomas of the breast. Turk. Patoloji Derg..

[47] TM P (2015). Macrophageal infiltration and microvessel density in laryngeal carcinoma: study of 52 cases. Acta Otorhinolaryngol. Italica.

[48] S LP (2018). Endothelial Tie1-mediated angiogenesis and vascular abnormalization promote tumor progression and metastasis. J. Clin. Investig..

[49] Z Z (2018). Cancer-derived exosomal miR-25-3p promotes pre-metastatic niche formation by inducing vascular permeability and angiogenesis. Nat. Commun..

[50] K F-K, Ø F, M T, CE N-X (2018). B7-H3 in cancer—beyond immune regulation. Trends Cancer.

[51] Y L (2017). B7-H3 promotes the migration and invasion of human bladder cancer cells via the PI3K/Akt/STAT3 signaling pathway. J. Cancer.

[52] B J (2016). The co-stimulatory molecule B7-H3 promotes the epithelial-mesenchymal transition in colorectal cancer. Oncotarget.

[53] DW L, G. C, WJ K, DV G, N F (1989). Vascular endothelial growth factor is a secreted angiogenic mitogen. Science.

[54] E T (1989). Vascular endothelial growth factor: a new member of the platelet-derived growth factor gene family. Biochem. Biophys. Res. Commun..

[55] Y T, Y K, CD B, KR C, LM E (1995). Expression of vascular endothelial growth factor and its receptor, KDR, correlates with vascularity, metastasis, and proliferation of human colon cancer. Cancer Res..

[56] LM E, Y T, W L, RM S (2000). Vascular endothelial growth factor in human colon cancer: biology and therapeutic implications. Oncologist.

[57] H L (2017). DHX32 promotes angiogenesis in colorectal cancer through augmenting β-catenin signaling to induce expression of VEGFA. EBioMedicine.

[58] C D (2017). Elevated Gab2 induces tumor growth and angiogenesis in colorectal cancer through upregulating VEGF levels. J. Exp. Clin. Cancer Res..

[59] Ferrara N, Adamis AP (2016). Ten years of anti-vascular endothelial growth factor therapy. Nat. Rev. Drug Disco..

[60] Jayson GC, Kerbel R, Ellis LM, Harris AL (2016). Antiangiogenic therapy in oncology: current status and future directions. Lancet.

[61] S W, Z L, L W, X Z (2009). NF-kappaB signaling pathway, inflammation and colorectal cancer. Cell. Mol. Immunol..

[62] SP T, AW G (2008). NF-kappa B: a new player in angiostatic therapy. Angiogenesis.

[63] A M (2011). IGF-1 and PDGF-bb suppress IL-1β-induced cartilage degradation through down-regulation of NF-κB signaling: involvement of Src/PI-3K/AKT pathway. PloS ONE.

[64] P S (2012). Aloe emodin inhibits colon cancer cell migration/angiogenesis by downregulating MMP-2/9, RhoB and VEGF via reduced DNA binding activity of NF-κB. Eur. J. Pharm. Sci. Off. J. Eur. Federation Pharm. Sci..

