# Supplementary material for: B7-H3 promotes colorectal cancer angiogenesis through activating the NF-κB pathway to induce VEGFA expression
Source: Cell Death Dis. 2020 Jan 23;11(1):55. doi: 10.1038/s41419-020-2252-3 (PMC6978425; doi:10.1038/s41419-020-2252-3)
Supplement: Supplementary file 1 — Supplementary Figure legends [file 41419_2020_2252_MOESM1_ESM.doc]

**Supplementary Figure legends**

**Supplementary Figure S1. The expression of B7-H3 and CD31 was associated with TNM stages and** **lymph node metastasis in tissue samples of CRC patients. a**, **b** B7-H3 (**a**) and CD31 (**b**) protein expression based on their staining index in in CRC specimens at different clinical stages. **c**, **d** B7-H3 (**c**) and CD31 (**d**) protein expression based on their staining index in CRC specimens with or without lymph node metastasis. The data represent the means ± SEM. ***P<0.001.

**Supplementary Figure S2. B7-H3 expression level in stable shB7-H3 CRC cell lines.** **a** The protein expression of B7-H3 in CRC cells treated with three B7-H3 siRNAs was detected by Western blot. **b**, **c** The mRNA expression of B7-H3 in HCT116 (**b**) and RKO (**c**) stable cell lines with B7-H3 inhibition (shB7-H3) or their control cell lines (sh-NC) was detected by RT-qPCR. **d** The protein level of soluble B7-H3 in sh-NC or shB7H3 cells were detected by ELISA. **e** The cell activity of HUVECs was examined by CCK-8 assays after HUVECs were treated with conditioned medium (CM) from sh-NC or shB7H3 cells. The data represent the means ± SEM. NS, no significant difference. **P<0.01; ***P<0.001.

**Supplementary Figure S3.** **B7-H3 overexpression promoted migration, invasion and tube formation of HUVECs. a**, **b** The mRNA expression of B7-H3 in HCT116 (**a**) and RKO (**b**) stable cell lines overexpressing B7-H3 (B7-H3) or their control cell lines (EV). **c** The protein expression of soluble B7-H3 in HCT116 and RKO stable cell lines overexpressing B7-H3 (B7-H3) or their control cell lines (EV). **d**, **e** Cell migration (**d**) and invasion (**e**) in HUVECs were examined by transwell assays after HUVECs were plated and treated with CM from EV cells or B7-H3 cells. Scale bar, 100 μm. One representative result from three reproducible experiments is shown. **f** Effect of CM from EV cells or B7-H3 cells on tube formation in HUVECs. Scale bar, 100 μm. One representative result from three reproducible experiments is shown. **g** The cell activity of HUVECs was examined by CCK-8 assays after HUVECs were treated with conditioned medium (CM) from EV or B7-H3 cells. The data represent the means ± SEM. NS, no significant difference. **P<0.01; ***P<0.001.

**Supplementary Figure S4. CRC cells treated with recombinant human B7-H3 (rB7-H3) promoted migration, invasion and tube formation of HUVECs. a**, **b** Cell migration (**a**) and invasion (**b**) in HUVECs were examined by transwell assays after HUVECs were plated and treated with conditioned medium (CM) from CRC cells treated with rB7-H3. Scale bar, 100 μm. One representative image from three reproducible experiments is shown. Migrated and invaded HUVEC numbers are shown in the bar graph. **c** Effect of CM from CRC cells treated with rB7-H3 on tube formation in HUVECs. Scale bar, 100 μm. The number of tubes counted is shown in the bar graph. One representative result from three reproducible experiments is shown. The data represent the means ± SEM. *P<0.05; **P<0.01; ***P<0.001.

**Supplementary Figure S5. B7-H3 overexpression promoted the expression of VEGFA in CRC cells.** **a** The expression of angiogenesis-related genes was detected by RT-qPCR in B7-H3 HCT116 and RKO cells. **b** The protein expression of VEGFA, bFGF and PDGF-BB in conditioned medium (CM) from sh-NC cells or shB7-H3 cells was examined by ELISA. **c** The protein expression of VEGFA, bFGF and PDGF-BB in conditioned medium (CM) from EV or B7-H3 CRC cell lines was examined by ELISA. **d** Western blot analysis of B7-H3 and VEGFA in EV or B7-H3 CRC cell lines. β-actin served as a loading control. **e** Immunofluorescence staining with VEGFA was performed in sh-NC and shB7-H3 CRC cell lines (red, VEGFA; blue, DAPI nuclear staining). One representative image from five repeats is shown. Scale bar, 100 μm. **f** Immunofluorescence staining for VEGFA was performed in EV or B7-H3 CRC cell lines (red, VEGFA; blue, DAPI nuclear staining). One representative image from five repeats is shown. Scale bar, 100 μm. **g** The protein expression of VEGFA in CRC cells after treatment with or without rB7-H3 was analyzed by ELISA. **h** The protein expression of VEGFA in CRC cells after treatment with or without rB7-H3 was analyzed by Western blot. The data represent the means ± SEM. NS, no significant difference; *P<0.05; **P<0.01; ***P<0.001.

**Supplementary Figure S6. Recombinant VEGFA (rVEGFA) reversed the effect of CM from shB7-H3 on cell migration, invasion and tube formation.**

**a**, **b** Cell migration (**a**) and invasion (**b**) in HUVECs were examined by transwell assays after HUVECs were co-treated with CM from sh-NC cells or shB7-H3 cells and IgG or recombinant VEGFA (rVEGFA). Scale bar, 100 μm. One representative result from three reproducible experiments is shown. **c** The tube formation of HUVECs co-treated with CM from sh-NC cells or shB7-H3 cells and IgG or rVEGFA was examined. Scale bar, 100 μm. One representative result from three reproducible experiments is shown.

**Supplementary Figure S7. VEGFA siRNA or neutralizing antibody reversed the effect of CM from B7-H3-overexpressing CRC cells on cell migration, invasion and tube formation.**

**a, b** The mRNA level of VEGFA in HCT116 (**a**) and RKO (**b**) cells treated with VEGFA siRNA or negative control (NC) was detected by RT-qPCR. **c** Western blot analysis of VEGFA in HCT116 and RKO cells treated with VEGFA siRNA or NC. β-actin served as a loading control. **d**, **e** Cell migration (**d**) and invasion (**e**) in HUVECs were examined by transwell assays after HUVECs were co-treated with CM from EV cells or B7-H3 cells and siRNA negative control (NC) or VEGFA siRNA. Scale bar, 100 μm. One representative result from three reproducible experiments is shown. **f** The tube formation of HUVECs co-treated with CM from EV cells or B7-H3 cells and NC or VEGFA siRNA was examined. Scale bar, 100 μm. One representative result from three reproducible experiments is shown. **g**, **h** Cell migration (**g**) and invasion (**h**) in HUVECs were examined by transwell assays after HUVECs were co-treated with CM from EV cells or B7-H3 cells and a VEGFA neutralizing antibody (antiVEGFA). Scale bar, 100 μm. One representative result from three reproducible experiments is shown. Migrated and invaded HUVEC numbers are shown in the bar graph. **i** The tube formation of HUVECs co-treated with CM from EV cells or B7-H3 cells and a VEGFA neutralizing antibody (antiVEGFA) was examined. Scale bar, 100 μm. The number of tubes counted is shown in the bar graph. One representative result from three reproducible experiments is shown. The data represent the means ± SEM. *P<0.05; **P<0.01; ***P<0.001.

**Supplementary Figure S8. B7-H3 promoted angiogenesis** **through the NF-κB pathway. a, b** The expression of genes downstream of the NF-κB pathway was detected by RT-qPCR in shB7-H3 (**a**) and B7-H3 (**b**) CRC cells. **c** The protein expression of p-p65 in CRC cells after treatment with or without rB7-H3 was analyzed by Western blot. **d**, **e** Cell migration (**d**) and invasion (**e**) in HUVECs were examined by transwell assays after HUVECs were co-treated with CM from EV cells or B7-H3 cells and BAY11-7082. Scale bar, 100 μm. One representative result from three reproducible experiments is shown. **f** The tube formation of HUVECs co-treated with CM from EV cells or B7-H3 cells and BAY11-7082 was examined. Scale bar, 100 μm. One representative result from three reproducible experiments is shown. The data represent the means ± SEM. *P<0.05; **P<0.01; ***P<0.001.

**Supplementary Figure S9. B7-H3 promoted angiogenesis in Matrigel plugs in vivo. a** Images of the subcutaneous tumors formed by sh-NC-HCT116 and shB7-H3-HCT116 cells. N = 5. **b**, **c** Quantification of the size (**b**) and weight (**c**) of subcutaneous tumors formed by sh-NC-HCT116 and shB7-H3-HCT116 cells. **d** Images of subcutaneous tumors formed by EV-HCT116 and B7-H3-HCT116 cells. N = 5. **e**, **f** Quantification of the size (**e**) and weight (**f**) of subcutaneous tumors formed by EV-HCT116 and B7-H3-HCT116 cells. NS, no significant difference. **g** Representative IHC images of subcutaneous tumors formed by sh-NC-HCT116 and shB7-H3-HCT116 cells. Scale bars, 100 μm. N = 5. **h** Representative IHC images of subcutaneous tumors formed by EV-HCT116 and B7-H3-HCT116 cells. Scale bars, 100 μm. N = 5.

**Supplementary Figure S10. B7-H3 promoted angiogenesis in Matrigel plugs through the NF-κB/VEGFA pathway in vivo.**  **a**, **b** Quantification of the size (**a**) and weight (**b**) of subcutaneous tumors formed by B7-H3-HCT116 cells treated with BAY11-7082 at a dose of 6 mg/kg every other day. **c**, **d** Quantification of the size (**j**) and weight (**k**) of subcutaneous tumors formed by B7-H3-HCT116 cells treated with bevacizumab at a dose of 1 mg/kg twice per week. e Representative IHC images of subcutaneous B7-H3-HCT116 tumors treated with BAY11-7082. Scale bars, 100 μm. N = 5. **f** Representative IHC images of subcutaneous B7-H3-HCT116 tumors treated with bevacizumab. Scale bars, 100 μm. N = 5. **g**, **h** Quantification of the size (**g**) and weight (**h**) of subcutaneous tumors formed by B7-H3-HCT116 cells co-treated with 3E8 (5 mg/kg) and BAY11-7082 (6 mg/kg) or bevacizumab (1 mg/kg). N = 5. **i** Representative IHC images of subcutaneous B7-H3-HCT116 tumors co-treated with 3E8 (5 mg/kg) and BAY11-7082 (6 mg/kg) or bevacizumab (1 mg/kg). Scale bars, 100 μm. N = 5. *P<0.05; **P<0.01; ***P<0.001.

**Supplementary Figure S11.** **Schematic diagram of the mechanism in this study.** B7-H3 promotes angiogenesis via activating NF-κB/VEGFA axis in CRC.
