# Supplementary material for: B7-H3 promotes colorectal cancer angiogenesis through activating the NF-κB pathway to induce VEGFA expression
Source: Cell Death Dis. 2020 Jan 23;11(1):55. doi: 10.1038/s41419-020-2252-3 (PMC6978425; doi:10.1038/s41419-020-2252-3)
Supplement: Supplementary file 2 — Supplementary Table S1. [file 41419_2020_2252_MOESM2_ESM.doc]

**Supplementary Table S1. Clinical characteristics of patients**

| CRC patients | Number |
| --- | --- |
| NO. of patients | 125 |
| Gender |  |
| Male | 73 |
| Female | 52 |
| Age (years) |  |
| Mean | 60.68 |
| Range | 26-81 |
| Tumor location |  |
| Colon | 74 |
| Rectun | 51 |
| TNM stage |  |
| I-II | 66 |
| III-IV | 59 |
