# Supplementary material for: B7-H3 promotes colorectal cancer angiogenesis through activating the NF-κB pathway to induce VEGFA expression
Source: Cell Death Dis. 2020 Jan 23;11(1):55. doi: 10.1038/s41419-020-2252-3 (PMC6978425; doi:10.1038/s41419-020-2252-3)
Supplement: Supplementary file 3 — Supplementary Table S2. [file 41419_2020_2252_MOESM3_ESM.doc]

**Supplementary Table S2. Antibodies for Western bolt in this study**

| Antibody Name | Details |
| --- | --- |
| JNK | CST, #9252, 1:1000 |
| p-JNK | CST, #9251, 1:1000 |
| ERK | CST, #4695, 1:1000 |
| p-ERK | CST, #5726, 1:1000 |
| AKT | CST, #4691, 1:1000 |
| p-AKT | CST, #4060, 1:2000 |
| STAT3 | CST, #9139, 1:1000 |
| p-STAT3 | CST, #9145, 1:2000 |
| NF-κB p65 | Beyotime, #AN365, 1:500 |
| p-NF-κB-p65 | Beyotime, #AN371, 1:1000 |
| human B7-H3 | R&D Systems, #AF1027, 1:250 |
| β-actin | Immunoway Biotechnology, #YM3028, 1:5000 |
| VEGFA | Proteintech, #19003-1-AP, 1:1000 |
