# Supplementary material for: B7-H3 promotes colorectal cancer angiogenesis through activating the NF-κB pathway to induce VEGFA expression
Source: Cell Death Dis. 2020 Jan 23;11(1):55. doi: 10.1038/s41419-020-2252-3 (PMC6978425; doi:10.1038/s41419-020-2252-3)
Supplement: Supplementary file 4 — Supplementary Table S3. [file 41419_2020_2252_MOESM4_ESM.doc]

**Supplementary Table S3. Primers for RT-qPCR in this study**

| Primer Name | Primer Sequence(5’-3’） |
| --- | --- |
| hsa-VEGFA-Forward | AGGGCAGAATCATCACGAAGT |
| hsa-VEGFA-Reverse | AGGGTCTCGATTGGATGGCA |
| hsa-VEGFB-Forward | GAGATGTCCCTGGAAGAACACA |
| hsa-VEGFB-Reverse | GAGTGGGATGGGTGATGTCAG |
| hsa-VEGFC-Forward | ATGTGTGTCCGTCTACAGATGT |
| hsa-VEGFC-Reverse | GGAAGTGTGATTGGCAAAACTGA |
| hsa-VEGFD-Forward | TCCCATCGGTCCACTAGGTTT |
| hsa-VEGFD-Reverse | AGGGCTGCACTGAGTTCTTTG |
| hsa-VEGFE-Forward | ATTCACAGCCCAAGGTTTCCT |
| hsa-VEGFE-Reverse | GGGTCTTCAAGCCCAAATCTT |
| hsa-PGF-Forward | GAACGGCTCGTCAGAGGTG |
| hsa-PGF-Reverse | ACAGTGCAGATTCTCATCGCC |
| hsa-bFGF-Forward | AGAAGAGCGACCCTCACATCA |
| hsa-bFGF-Reverse | CGGTTAGCACACACTCCTTTG |
| hsa-PDGFB-Forward | CTCGATCCGCTCCTTTGATGA |
| hsa-PDGFB-Reverse | CGTTGGTGCGGTCTATGAG |
| hsa-WNT7B-Forward | CACAGAAACTTTCGCAAGTGG |
| hsa-WNT7B-Reverse | GTACTGGCACTCGTTGATGC |
| hsa-MMP2-Forward | TACAGGATCATTGGCTACACACC |
| hsa-MMP2-Reverse | GGTCACATCGCTCCAGACT |
| hsa-CXCL9-Forward | CCAGTAGTGAGAAAGGGTCGC |
| hsa-CXCL9-Reverse | AGGGCTTGGGGCAAATTGTT |
| hsa-THBS1-Forward | AGACTCCGCATCGCAAAGG |
| hsa-THBS1-Reverse | TCACCACGTTGTTGTCAAGGG |
| hsa-B7-H3-Forward | ACAGGGCAGCCTATGACATT |
| hsa-B7-H3-Reverse | CTGCATTCTCCTCCTCACAG |
| hsa-β-actin-Forward | AAGGAGCCCCACGAGAAAAAT |
| hsa-β-actin-Reverse | ACCGAACTTGCATTGATTCCAG |
| hsa-IL8-Forward | TTTTGCCAAGGAGTGCTAAAGA |
| hsa-IL8-Reverse | AACCCTCTGCACCCAGTTTTC |
| hsa-COX-2-Forward | CTGGCGCTCAGCCATACAG |
| hsa-COX-2-Reverse | CGCACTTATACTGGTCAAATCCC |
| hsa-Bcl2-Forward | GGTGGGGTCATGTGTGTGG |
| hsa-Bcl2-Reverse | CGGTTCAGGTACTCAGTCATCC |
| hsa-CyclinD1-Forward | GCTGCGAAGTGGAAACCATC |
| hsa-CyclinD1-Reverse | CCTCCTTCTGCACACATTTGAA |
