# Supplementary material for: B7-H3 promotes colorectal cancer angiogenesis through activating the NF-κB pathway to induce VEGFA expression
Source: Cell Death Dis. 2020 Jan 23;11(1):55. doi: 10.1038/s41419-020-2252-3 (PMC6978425; doi:10.1038/s41419-020-2252-3)
Supplement: Supplementary file 16 — DECLARATION OF CONTRIBUTIONS TO ARTICLE [file 41419_2020_2252_MOESM16_ESM.pdf]

DECLARATION OF CONTRIBUTIONS TO ARTICLE **ADMC**

Manuscript Number:	Journal Name:	
CDDIS-19-3148	*Cell Death & Disease*	(the 'Journal')

Proposed Title of the Contribution:

B7-H3 promotes colorectal cancer angiogenesis through activating the NF-κB pathway to induce VEGFA expression (the 'Contribution)

Author(s):

Ruoqin Wang, Yanchao Ma, Shenghua Zhan, Guangbo Zhang, Lei Cao, Xueguang Zhang, Tongguo Shi, Weichang Chen (the 'Authors')

For all *CDDis* articles, each person named as an author in the published version must be able to show he or she has contributed substantially to the article.

Authorship credit should be based on 1) substantial contributions to conception and design, acquisition of data, or analysis and interpretation of data; 2) drafting the article or revising it critically for important intellectual content; and 3) final approval of the version to be published. Authors should meet conditions 1, 2 and 3.

Any person who cannot be shown to have made a substantial contribution to the article cannot be listed as an author in the final version. The name of any person who is deemed to have made a minor contribution can, however, appear in the Acknowledgments section of the article.

Please complete the table below to indicate the contributions of all named authors to the manuscript.

Author Full Name:	Specification of Contribution to the Manuscript:
Ruoqin Wang	acquisition of data, drafting the article
Yanchao Ma	acquisition of data, analysis and interpretation of data
Shenghua Zhan	acquisition of data
Guangbo Zhang	analysis and interpretation of data
Lei Cao	analysis and interpretation of data
Xueguang Zhang	analysis and interpretation of data
Tongguo Shi	conception and design,revising it critically for important intellectual content
Weichang Chen	conception and design, final approval of the version to be published

Please complete the table below to indicate the contributions of all named authors to the figures.

Figure 1:
Ruoqin Wang, Yanchao Ma, Shenghua Zhan,Tongguo Shi

Figure 2:
Ruoqin Wang, Yanchao Ma, Lei Cao,

Figure 3:
Ruoqin Wang, Guangbo Zhang, Xueguang Zhang, Tongguo Shi

Figure 4:
Ruoqin Wang, Tongguo Shi, Weichang Chen

Figure 5:
Ruoqin Wang, Guangbo Zhang, Weichang Chen

Figure 6:
Ruoqin Wang, Tongguo Shi, Weichang Chen

Signed for and on behalf of the Author(s):	Print Name:	Date:
Tongguo Shi	Tongguo Shi	December 17, 2019
